# Research Progress on G-Quadruplexes in Human Telomeres and Human Telomerase Reverse Transcriptase (hTERT) Promoter

**DOI:** 10.1155/2022/2905663

**Published:** 2022-06-06

**Authors:** Wei Gu, Zihan Lin, Shengchao Zhao, Guanzhen Wang, Ziyi Shen, Wei Liu, Yi Cai, Kaibo Wang, Chunpeng Craig Wan, Tingdong Yan

**Affiliations:** ^1^School of Life Sciences, Shanghai University, Shanghai 200444, China; ^2^University and College Key Lab of Natural Product Chemistry and Application in Xinjiang, School of Chemistry and Environmental Science, Yili Normal University, Yining 835000, China; ^3^Guangzhou Municipal and Guangdong Provincial Key Laboratory of Molecular Target & Clinical Pharmacology, The NMPA and State Key Laboratory of Respiratory Disease, School of Pharmaceutical Sciences and the Fifth Affiliated Hospital, Guangzhou Medical University, Guangzhou 511436, China; ^4^Jiangsu Key Laboratory of Bioactive Natural Product Research and State Key Laboratory of Natural Medicines, China Pharmaceutical University, Nanjing 210009, China; ^5^Jiangxi Key Laboratory for Postharvest Technology and Nondestructive Testing of Fruits & Vegetables, College of Agronomy, Jiangxi Agricultural University, Nanchang 330045, China

## Abstract

The upregulation telomerase activity is observed in over 85-90% of human cancers and provides an attractive target for cancer therapies. The high guanine content in the telomere DNA sequences and the hTERT promoter can form G-quadruplexes (G4s). Small molecules targeting G4s in telomeres and hTERT promoter could stabilize the G4s and inhibit hTERT expression and telomere extension. Several G4 ligands have shown inhibitory effects in cancer cells and xenograft mouse models, indicating these ligands have a potential for cancer therapies. The current review article describes the concept of the telomere, telomerase, and G4s. Moreover, the regulation of telomerase and G4s in telomeres and hTERT promoter is discussed as well. The summary of the small molecules targeting G4s in telomeric DNA sequences and the hTERT promoter will also be shown.

## 1. Telomeres and Telomerase

Telomeres are dynamic nucleoprotein structures situated at the terminus of linear chromosomes and are the critical parts of chromosome structures in eukaryotic cells. Telomeres consist of highly conserved tandem DNA repeats which are linked with certain telomeric proteins. The repetitive sequences are conserved in over 90 eukaryotes, including mammals [1]. Binding of telomeric sequence-specific binding protein and their associated factors form a cap at the end of the linear chromosome. TRF1, TRF2, RAP1, TIN2, TPP1, and POT1 bind with telomeres, which form shelterin complex to prevent telomeres from end-end fusion, degradation, and recombination. TRF1 and TRF2 directly interact with double-stranded TTAGGG repeats, while POT1 interacts with 3′ single-stranded overhangs [2, 3]. The telomeric 3′ single-stranded overhangs form t-loop regulated by CDK phosphorylation of TRF2 during the cell cycle [4]. DNA replication follows two processes which are semiconservative replication and semidiscontinuous replication. In this procedure, DNA polymerase synthesizes a new DNA chain in the 5′ to 3′ direction with the help of tRNA primers. After the removal of tRNA primers, about 50-200 bp of telomeres DNA sequence will be lost at the 5′ end of the newly synthesized DNA strand [3, 5]. In stem cells and germ cells, the length of telomeres is often longer than that of somatic cells, which is because these cells have high telomerase activity and the telomerase is naturally silenced in the majority of somatic cells [6]. The literature indicates that telomerase is transiently expressed in the S phase during the normal cell division cycle to maintain the 3′ overhang of telomeres. When the 3′ overhang is destroyed or shortens to a critical limit, the telomere-binding proteins fail to protect the chromosomal ends, and this will lead to the uncapping of telomere and induction of DNA damage responses [6]. Furthermore, this will lead to cell cycle arrest and induction of cellular senescence and apoptosis through p53 and other signaling pathways [7–11].

**Figure 1:**
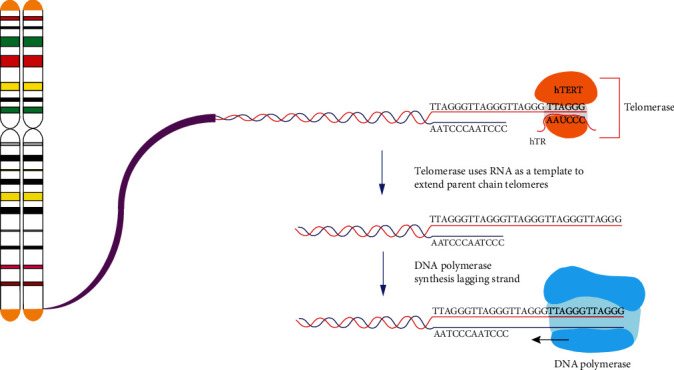
Telomere elongation by telomerase. Telomerase reverse transcriptase synthesizes telomeres DNA sequence which requires hTER as an RNA template and DNA polymerase to complete telomere elongation [1, 13].

Telomerase is a ribonucleoprotein complex with reverse transcriptase activity, and its function is to extend the length of the telomere. Telomerase activity can be detected in tissues from the fetal to the newborn. But after birth, the telomerase activity in somatic cells is lost. Telomerase is expressed in the ovary and testis in the reproductive system, but not in mature sperm and ovum [12]. Telomerase contains two essential subunits, the protein subunit human telomerase reverse transcriptase (hTERT) and the RNA subunit which is human telomerase RNA (hTERC or hTER). hTER contains a templating region (5′-CUAACCCUAAC-3′) that is necessary for the hTERT to copy the newly synthesized telomere sequence at the terminus of the chromosome [13] (Figure 1). The activity of telomerase is strictly regulated, which affects the cell fate by maintaining the stability of the telomere structure. If the activity of telomerase is only half of the normal level, it will lead to the shortening of telomeres [14]. The upregulation of telomerase activity in cancer cells is necessary for the telomere maintenance in tumor cells and underlies the ability of cancer cells to divide continuously. Interestingly, the telomere length of cancer cells is often shorter than normal cells, perhaps because the cells divide rapidly at an early stage of tumor development that exceeds the ability of telomerase synthesis [14]. About 85-90% of cancer cells obtained the ability of self-renewal and proliferation through activation of telomerase. Besides, there are about 5-10% of cancer cells use alternative lengthening of telomeres (ALT) to maintain telomeres [15]. Bodnar et al. reported that transferring of exogenous hTERT can exceed their normal lifespan and maintain normal human cells in a phenotypically youthful state, which could have important applications in telomere-related diseases and antiaging research [16]. Telomerase, as the key molecule to maintain telomeres, is of great significance for the regulation of cell senescence and cell division. A large number of studies have focused on the regulation of telomerase activity, hoping to develop new strategies to achieve targeted telomerase inhibition for cancer therapy and telomerase activation for the intervention in human aging.

## 2. Regulation of Human Telomerase

hTERT and hTER are two main targets for telomerase regulation. hTERT synthesizes the telomere DNA sequence using the hTER RNA motif as a template. For the RNA template sequence in hTER, the corresponding antisense oligonucleotide can be combined with it to inhibit the activity of telomerase. In somatic cells, telomerase lost function because of hTERT gene silencing, not hTER. hTER is universally expressed in all tissues and not a limiting factor for telomerase activity. It is noteworthy that hTER is highly expressed in cancer cells and germ cells to maintain the high telomerase [13]. Because telomerase is so important in human cancer and aging, numerous studies focus on the regulation of telomerase. The regulation of telomerase is a complex biological process and involves regulation of hTERT expression, regulation of hTERT translation, regulation of hTERT by epigenetic means, chromosome rearrangement, and miRNAs [17–19]. hTERT promoter covers about 3.5 kb upstream of the transcription start site, and there are multiple transcription factor binding sites such as SP1 and c-MYC that have been identified by methods such as reporter assay, ChIP, and EMSA. Enhancers located in the -20 kb upstream of the transcription start site (TSS) region also play important role in the regulation of hTERT [17–19]. Recently, studies reported that there are two gain-of-function mutations (C228T and C250T) in the hTERT promoter region, which cause telomerase activation and drive tumorigenesis in human cancer such as melanoma, glioma, liver cancer, and urothelial cancer [20–23], which provided an attractive target for cancer therapy. It has been proved that there are 12 consecutive G-tracts in the promoter sequence from -100 to -168 form special G4 structures, which inhibit the expression of hTERT. Small molecules binding to G4 structure could decrease the expression of hTERT and inhibit cancer cell proliferation.

## 3. G-Quadruplex Structure and Its Regulation

**Figure 2:**
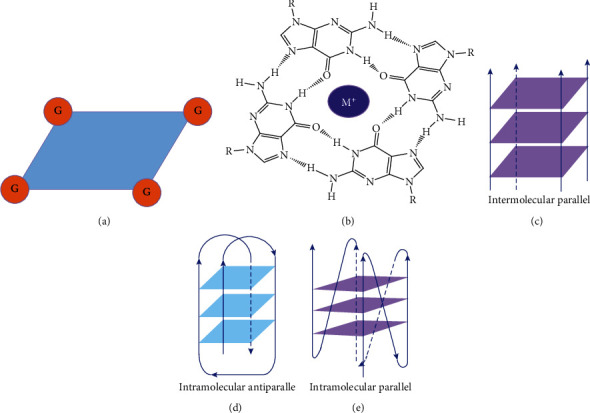
The structure of G-quartet and common G4 types. (a) Guanines form the planar structure of G-quartet. (b) G-quartet stabilized by hydrogen and cation like K^+^ and Na^+^. (c–e) Illustration of common G4 types [34].

G-quartet is a very stable planar structure that consists of four guanines by Hoogsteen bond to form hydrogen bonds and metal ions like K^+^ (Figures 2(a) and 2(b)). Following the literature, it is evident that K^+^ > Na^+^ > Li^+^ plays an important role in the maintenance of the G-quartet structure. Two or more *π*–*π* stacked G-quartets form G4 structure [24–26]. G4 is found in DNA and RNA sequences which are rich in GC bases. G4 is an atypical secondary structure of DNA, continuous G-rich DNA sequences containing multiple G-tracts are folded to form intramolecular G4 in single DNA, and double-stranded DNA can form interstrand G4 [26] (Figure 2(c)–2(e)). There is another atypical nucleic acid secondary structure named i-motif, formed by four cytosines that has been proved to exist in the double-stranded DNA sequence with GC bases rich. Further studies showed that the formation of i-motif and G4 is interdependent in human cells [27, 28]. There are many proposed methods for G4 research: the possibility of G4 formation is predicted and analyzed based on the base sequence of the target DNA fragment; the nucleic acid sequence designed and synthesized in vitro can be determined to form the G4 structure through physical and chemical methods such as circular dichroism, X-ray, nuclear magnetic resonance, and gel migration analysis; Thioflavin T can bind specifically to G4 and enhance Thioflavin T fluorescence; G4 dimer can enhance the fluorescence intensity of Thioflavin T dramatically as compared to monomeric G4 [29, 30]; it is a mature and common method to study the biological function of G4 through molecular cloning and luciferase reporter assay [31, 32]. G4s are not randomly distributed on the DNA sequence; it is found that G4s mainly exist in the promoter of the genes and the chromatin terminal telomere DNA by G4-specific antibodies and genome sequencing. Generally, the DNA sequence that forms the G-4 has a similar base arrangement. G_3-5_N_1-7_G_3-5_N_1-7_G_3-5_N_1-7_G_3-5_ is the general motif of the G4 structure, which can help us to predict and analyze the possible sequence of G4. Previously, a relatively simple algorithm was used to search for this consensus sequence in the genome sequence, and more than 300,000 possible DNA G4 sites were identified from the human genome. Recently, more than 700,000 potential G4 sites were identified in the human genome by G4 ChIP-seq. Interestingly, more than 40% of human gene promoter regions contain at least one G4 motif [33–35], suggesting the important function of G4 in the regulation of gene expression. Cancer is one of the most threatening diseases to human health, and its pathogenic mechanism is very complicated. Studies have found that many proteins with important functions are involved in tumorigenesis. If G4s are involved in the gene expression, studying the relationship between G4 and oncogene expression can help us to understand the mechanism of tumorigenesis and provide targets for cancer therapy. Myc is a proto-oncogene, as a transcription factor that participates in the regulation of cell proliferation, metabolism, apoptosis, and other important processes. Upexpression of Myc has been observed in up to 80% of solid tumors, such as gastric cancer, ovarian cancer, and breast cancer [36, 37]. In vivo, there is a sequence containing six G-tracts with different numbers of G formed G4 structure in the promoter of Myc, which could inhibit the expression of Myc. After treatment with G4 stabilizers such as TMPyP4, the expression of MYC was significantly reduced [38]. Not only in the proto-oncogene but also the promoter of some tumor suppressor genes may also form G4. In most cases, G4 downregulates gene expression by preventing transcription factors from binding to gene promoters or hindering the sliding of RNA polymerase along with DNA. However, G4 structure can not only inhibit gene expression but G4 formed in the promoter may promote gene expression. As a deubiquitinating enzyme, BAP1 is also considered an antioncogene. There is a G4 structure in the promoter of BAP1, and it upregulates the expression of BAP1, which in turn achieves the antitumor effect. In addition, in the promoter of relaxin and OCT, the G4 structure also promotes gene expression [39–41].

After G4 is formed in the DNA sequence, it does not become a stable structure on the genome. There are more than 20 helicases such as FANCJ and Pif1 that can bind and untie G4s in the cells. Pif1 can untie G4s after the small-molecule G4 ligands bind to G4s [42]. This suggests that small-molecule ligands targeting G4s in the promoter of genes may not achieve the expected effect of gene regulation under the action of helicases. In addition to the gene promoters, another important region where G4s exist is the chromatin terminal telomeres DNA. The G4s were first discovered in the G-rich single-stranded G-tail structure in the 3′ end of the telomere DNA. Telomeres play an essential role in tumor proliferation, so numerous studies have linked G4s to the inhibition of telomere elongation, through G4 ligands to stabilize the structure of telomere terminal G4 and inhibit the proliferation of cancer cells [43–45].

## 4. G-Quadruplex Structures in Telomeres and hTERT Promoter

**Figure 3:**
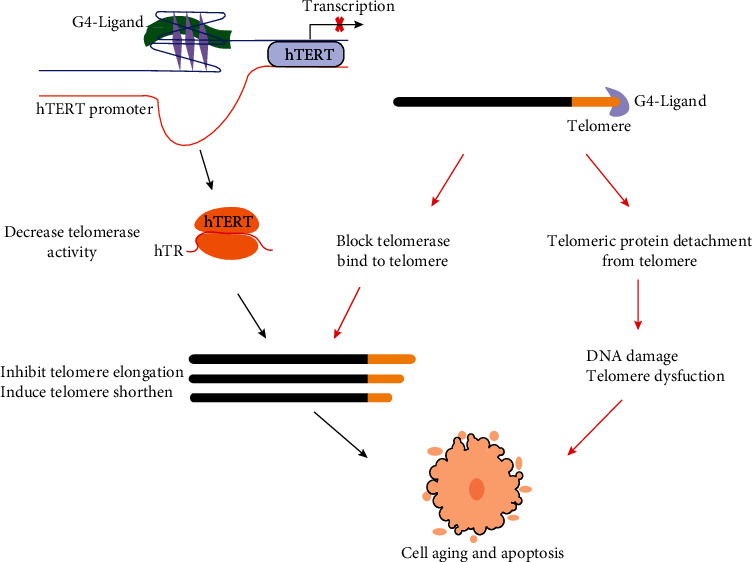
The mechanism of telomere and telomerase promoter G4 ligands inhibit cancer cells growth. The black arrows indicate that G4 ligands targeting hTERT promotor G4 shorten telomere, and this is a slow effect that induces cell death. The telomeric G4 ligand bind telomere could quickly inhibit the growth of cancer cells which is indicated by red arrows [11, 26].

G4 structures can be formed in telomere DNA sequence and hTERT promoter, which suggests G4 is a potential target to inhibit telomere elongation and telomerase activity. The telomere terminal repeat sequence TTAGGG is highly rich in guanine, and there are often G4s at the telomere, especially in 3′ overhang. Generally, telomerase needs helicase to untie the G4 structure before the elongation of the telomere. If some G4 ligands bind and stabilize the G4s at the end of telomeres, the helicase cannot perform its function, and the G4s blocking the telomerase bind with telomere. In the subsequent cell division, the telomere length will gradually shorten and eventually trigger replication senescence [11, 46] (Figure 3). Such drugs can also cause the depolymerization of the telomere terminal protein complex. When telomere is destroyed, the telomere-binding proteins can no longer protect the chromosome end, and this will induce DNA damage response and lead to cellular senescence and apoptosis [47]. This method has attracted a lot of attention and maybe a promising treatment for targeting telomeres because this kind of drug can directly affect telomeres, and they have a faster effect which acts as a revival of telomerase activity.

**Figure 4:**
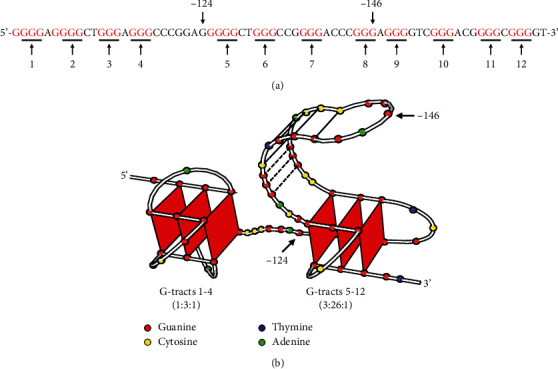
The G4s in hTERT core promotor. (a) The 12 consecutive G-tracts in the hTERT promoter can form G4s. (b) The hairpin structure model of G4s in hTERT core promoter [52].

Another method is to stabilize the G4 structure in the hTERT promoter and reduce the expression of hTERT, thereby reducing the telomerase activity and preventing cells from synthesizing telomere end repeat sequences and leading to aging and apoptosis (Figure 3). The hTERT promoter G4 is a potential drug target, and further studying its structure will be helpful for drug development. Currently, there are two widely accepted models for hTERT promoter G4s. In the first model, Monsen et al. and Chaires et al. provided that there is a three-parallel G4 conformation in the wild-type hTERT promoter. The triple parallel quadruple structure consists of 1-4 G-tracts, 5-8 G-tracts, and 9-12 G-tracts, folding into a compact stacked three-G-4 conformation [48, 49]. The second model suggested that the 5-8 G-tracts alone may not easily form G4, but the 5-8 G-tracts and the 9-12 G-tracts form G4 and connected the 1-4 G beam by the 26 bp length hairpin structure. In the other words, the numbers 1-4 and 5, 6, 11, and 12 G-tracts formed two typical stable parallel G4 structures called G4 dimer in the hTERT promoter [50–52] (Figure 4). Both models believed that the 1-4 G-tracts alone forms a G4, which is a hybrid structure of parallel and antiparallel (3 + 1) [50]. The first model has been verified in various physical and chemical methods in vitro, and the second model has more support from drug experiments. Considering the complexity of the *in vivo* environment, the existing model of the G4s in the hTERT promoter may require more study to verify. The drugs targeting hTERT promoter G4 need to regulate gene expression to shorten the effect of telomeres and inhibit proliferation; it will take a long term to see the effects [53]. Although G4 ligand drugs have shown attractive prospects, both the telomere structure and the complexity of G4 require further study. Some small molecular G4 ligands have good antitumor effects in vitro and animal models, but the mechanism needs further study. Because of the lack of clinical research data to support, G4 ligands still have a long way to be used as a clinical drug.

## 5. Small Molecules Targeting G4s in Telomeres and hTERT Promoter

The cationic porphyrin TMPyP4 can bind and stabilize the G4s in the human telomere sequence, thereby hindering the binding of telomerase to the telomere. TMPyP4 shows the inhibitory effect on breast cancer and prostate cancer as well as decreases telomerase activity at the cellular and animal levels. However, TMPyP4 can also bind G4 in the promoter of c-Myc and inhibit the expression of c-Myc. c-Myc is a transcription factor of hTERT, so TMPyP4 can indirectly decrease the expression of hTERT by downregulation of c-Myc [54]. TMPyP4 leads to telomere DNA damage response and activates cellular senescence and apoptosis [55, 56]. Telomestatin can bind to telomere G4, showing both high quadruplex affinity and telomerase inhibitory potency. Telomestatin inhibits telomerase activity and tumor growth in a leukemia xenograft mouse model [57, 58]. BRACO19 is one of the most potent inhibitors of telomerase, with high specificity for telomeres G4 and induce telomere DNA damage and cellular senescence by inducing stable telomere G4, hindering terminal protein complex to detach. BRACO19 showed a strong inhibitory effect and short treatment time; it can have about 90% inhibitory effect in the uterine tumor xenograft mouse model [54, 59]. BRACO-19 suppresses proliferation and reduces telomerase activity on glioma cells and has no effect on normal human astrocytes, showing good selectivity for tumor cells. The excellent performance also makes BRACO19 one of the most promising drugs for clinical application [60]. Pyridostatin can stabilize telomere G4 and compete with POT1 for telomere binding. After POT1 dissociates from telomeres, it induces a DNA damage response, leading to the death of cancer cells [61]. RHPS4 is an acridine derivative, and it induces a strong telomere damage response and cell apoptosis by binding to telomere G4 to cause the dissociation of POT1 from the telomere. It can also reduce hTERT expression and inhibit telomerase activity, causing telomere length shortening. RHPS4 had a good inhibitory effect on tumor cell lines and xenograft mouse models and has almost nonspecific damage to nontumor tissues. In further drug combination studies, it was found that the combination of RHPS4 with paclitaxel showed a synergistic effect on uterine tumors [53, 62]. Although most cancers have acquired the ability to immortalize through the reexpression of telomerase, about 20% of tumor cells can be detected mutations in the hTERT promoter, and these mutations cause the upregulation of hTERT expression and telomerase activity [63]. Two highly abundant hotspot mutations, G>A mutations at 124 and 146 sites, were found in the gene-sequencing analysis in various tumor cells. Further studies confirmed that these two mutations inhibited the formation and stabilization of G4 which is formed by the numbers 5, 6, 11, and 12 G-tracts in the hTERT promoter, thereby upregulating hTERT expressed in tumor cells. The propylguanidino-acridine derivative GTC365 directly binds the G4 formed by the 5th, 6th, 11th, and 12th G tetrad fold in the hTERT promoter and reduces the expression of hTERT. GTC365 can also strengthen the stability of G4 and help G4 to inhibit hTERT expression, reducing telomere length and promoting cancer cellular senescence and apoptosis. In melanoma cells, the GTC365 shows the therapeutic potential of decreasing the hTERT expression. GTC365 inhibits tumor growth which is significantly better than BRACO19, especially when cancer cells are treated with low concentration [52]. RG260 contains benzoylphenylurea (BPU) which can bind to hairpin structure between quadruplets of promoter but lacks the acridine moiety of GTC365 that binds to the quadruples. RG260 targets the hairpin structure in the hTERT G4s, induces 9-12 G bundles to form a G4, and reduces hTERT expression. In prostate cancer, RG260 and its derivatives may induce oxidative stress and human prostate tumor cell apoptosis through mitochondrial damage, without harming normal human prostate epithelial cells. In subsequent *in vivo* xenografts, RG260 derivatives caused significant growth inhibition of prostate cancer [64]. Triazinel 12459 shows good therapeutic potential in lymphoma. On the one hand, inducing the formation of telomere G4 will cause the dysfunction of telomerase and further lead to telomere shortening and apoptosis. On the other hand, it can affect the splicing of hTERT mRNA and affect the telomerase activity by downregulating the hTERT mRNA expression [65, 66]. Thiazole orange is a G4 ligand. By modifying its side chain with different amino acids, it was found that various derivatives have strong inhibitory effects on telomerase activity in vitro. Among them, thiazole orange-spermine conjugate has a similar effect to BRACO19. But the role of these derivatives in cancer cells and mouse models needs further study [67]. BMPQ-1 with the 5H-pyrazolo [4,3-c] quinoline core has strong selectivity for the G4 at the end of the telomere and can stabilize G4 at the 3′ overhang of the telomere. BMPQ-1 can induce the formation of telomere G4, causing telomere dysfunction and DNA damage response, followed by cell senescence and apoptosis. The BMPQ-1 also showed good tumor-suppressing effects in lung, liver, and gastric cancer cells and human colon adenocarcinoma xenograft mouse models, even better than of the classical G4 ligand BRACO19 [68]. In traditional Chinese medicine, many natural compounds have been found to have the ability to stabilize G4 in telomeres and hTERT promoter, and the modified derivatives of these natural products have stronger effects. Natural compounds have the advantages of being extensive, easy to extract, and lower toxicity, so more effective G4 ligands can be found from there. Alkaloids, as a natural medicine research focus, are expected to provide stable G4 drug molecules. Berberine is isolated from the Chinese herbal medicine *Rhizoma Coptidis*, a natural isoquinoline alkaloid, which has anti-inflammatory, antibacterial, and anticancer effects. Berberine is also used in chronic diseases, protects cardiovascular issues, and lowers blood lipids and blood sugar levels [69]. Researchers found that berberine can inhibit the proliferation of several cancer cells such as cervical cancer and has little or no toxicity in normal cells. Berberine inhibits tumor growth by inducing and increasing G4 stability and decreasing telomerase activity. The berberine derivatives which are alkyl-substituted at positions C-9 and C-13 have strong specificity for telomere G4 [70–72]. In vitro physicochemical properties, studies on the ability of other natural alkaloids to induce and stabilize G4 have shown that the interaction between benzophenanthridine alkaloids and telomere G4s is stronger than isoquinoline alkaloids. However, there is a lack of research at the cell and animal levels. Further study needs to be done to find more effective pharmaceutical ingredients to stabilize G4 in telomeres and hTERT promoter from natural products [73, 74] (Table 1).

## 6. Conclusion and Perspectives

Numerous studies have confirmed that telomere and telomerase are associated with cancer, neurological diseases, aging, and other diseases. The upregulation of telomerase activity in cancer cells is necessary for the telomere maintenance in cancer cells and underlies the ability of cancer cells to divide continuously. Therefore, telomerase has become a potential target for cancer therapy. However, telomerase inhibition may also cause undesirable effects on stem cells and progenitor cells. Recent studies showed that two mutations in the hTERT promoter can upregulate hTERT expression in glioblastoma, melanoma, and liver cancer. These mutations abrogate the G4-folding process and result in activation of hTERT, suggesting a potential specific target for cancer therapy. G4 has been confirmed to exist in the hTERT promoter, so it is possible to target hTERT promoter G4 for therapy. Although several drugs targeting the G4 have achieved good results in vitro and mouse models, G4 ligand drugs are still a long way away from clinical application. The first problem that needs to be resolved is the specificity of the G4 ligand drugs. Some drugs targeting G4 in the promoter may bring us other side effects while suppressing tumors. The development of G4 drugs targeting telomeres and hTERT promoter with clinical application prospects not only has a strong effect on tumor cells but also has a specific binding target to avoid any unnecessary binding to other molecules for modulation of certain gene expressions. Resistance is an important factor limiting the use of most anticancer drugs. After treatment with G4 ligands, some cancer cells develop resistance and even increase the expression of telomerase [65]. The ligand targeting G4 in the hTERT promoter takes time to show the inhibition effect. Although it hinders the function of telomerase to lengthen telomeres, cancer cells continue to proliferate until the length of telomeres is shortened to the critical limit. Considering the different stages of the tumor, only the use of G4 ligand drugs may not be able to achieve the optimal antitumor effect. The combination of G4 ligand drugs with other clinical used drugs may achieve better therapeutic effects. With the improvement of drug design and the further study of the mechanism of G4s, new G4 ligand drugs that target telomeres and hTERT promoter will make progress in solving specificity, safety, and drug resistance, and breakthroughs will be made in clinical experiments soon.

## Figures and Tables

**Table 1:** Small molecules targeting G4s in telomeres and hTERT promoter.

Compound	Chemical structure	Main tumor cell type
GTC365	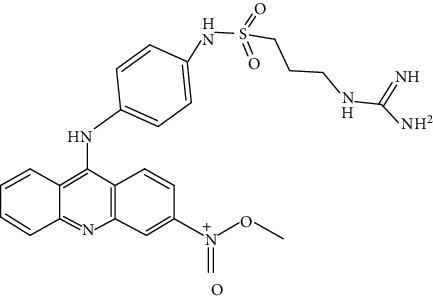	Melanoma [52]
TMPyP4	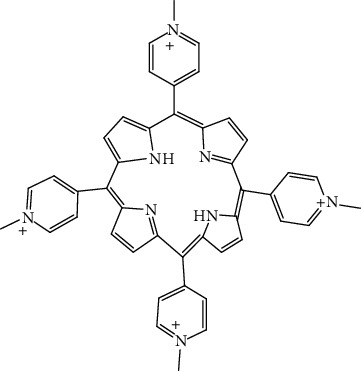	Mammary cancer, prostatic cancer [55]
Telomestatin	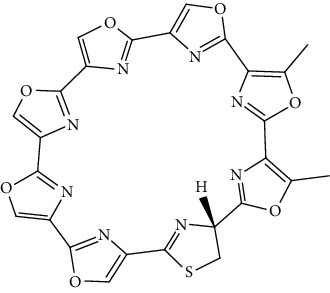	Lymphoma [59]
BRACO19	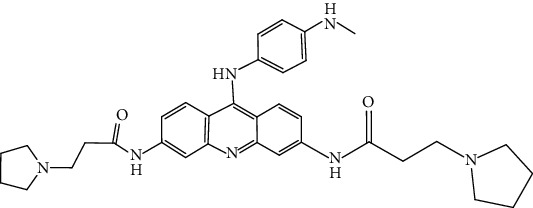	Uterine carcinoma [60], Glioma [61]
RHPS4	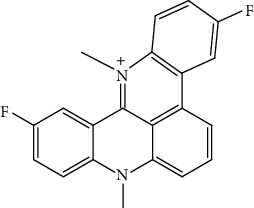	Breast carcinoma [63]
BMPQ-1	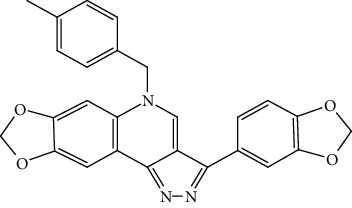	Lung cancer, liver cancer, and colon adenocarcinoma [69]
Berberine	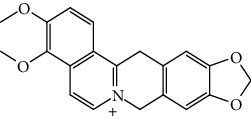	Cervical cancer, colorectal cancer [71, 72]

## Data Availability

The data used to support the findings of this study are available from the corresponding author upon request.
